# A comprehensive analysis of breast cancer microbiota and host gene expression

**DOI:** 10.1371/journal.pone.0188873

**Published:** 2017-11-30

**Authors:** Kevin J. Thompson, James N. Ingle, Xiaojia Tang, Nicholas Chia, Patricio R. Jeraldo, Marina R. Walther-Antonio, Karunya K. Kandimalla, Stephen Johnson, Janet Z. Yao, Sean C. Harrington, Vera J. Suman, Liewei Wang, Richard L. Weinshilboum, Judy C. Boughey, Jean-Pierre Kocher, Heidi Nelson, Matthew P. Goetz, Krishna R. Kalari

**Affiliations:** 1 Department of Health Sciences Research, Mayo Clinic, Rochester, Minnesota, United States of America; 2 Department of Center for Individualized Medicine, Mayo Clinic, Rochester, Minnesota, United States of America; 3 Department of Oncology, Mayo Clinic, Rochester, Minnesota, United States of America; 4 Department of Surgery, Mayo Clinic, Rochester, Minnesota, United States of America; 5 Department of Pharmaceutics, University of Minnesota, Minneapolis, MN, United States of America; 6 Department of Molecular Pharmacology & Experimental Therapeutics, Mayo Clinic, Rochester, Minnesota, United States of America; Indiana University, UNITED STATES

## Abstract

The inflammatory tumoral-immune response alters the physiology of the tumor microenvironment, which may attenuate genomic instability. In addition to inducing inflammatory immune responses, several pathogenic bacteria produce genotoxins. However the extent of microbial contribution to the tumor microenvironment biology remains unknown. We utilized The Cancer Genome Atlas, (TCGA) breast cancer data to perform a novel experiment utilizing unmapped and mapped RNA sequencing read evidence to minimize laboratory costs and effort. Our objective was to characterize the microbiota and associate the microbiota with the tumor expression profiles, for 668 breast tumor tissues and 72 non-cancerous adjacent tissues. The prominent presence of *Proteobacteria* was increased in the tumor tissues and conversely *Actinobacteria* abundance increase in non-cancerous adjacent tissues. Further, geneset enrichment suggests *Listeria spp* to be associated with the expression profiles of genes involved with epithelial to mesenchymal transitions. Moreover, evidence suggests *H*. *influenza* may reside in the surrounding stromal material and was significantly associated with the proliferative pathways: G2M checkpoint, E2F transcription factors, and mitotic spindle assembly. In summary, further unraveling this complicated interplay should enable us to better diagnose and treat breast cancer patients.

## Introduction

Cancer is a complex disease, where a multitude of genomic and physiological alterations occurring incessantly in the tumor tissue adds to the complexity of disease treatment and management[1]. The microenvironment in and around the tumor constitutes a variety of cell types, purportedly including microbiota. Pathophysiological changes occurring in the cells and in the microbial composition could have significant impact on the tumor growth [1–4]. While impactful discoveries have been made in cancer diagnosis and treatment by investigating the intricate shifts in the cellular and molecular biology of the tumor, microbial contributions to tumor growth remain unexplored. Diversity shifts in tumor microbiota have been observed in a variety of cancers including: prostate cancer [5], cervical cancer [6], colorectal cancer [7, 8], and lung cancer [6, 9], and breast cancer [10–12].

Breast cancer is one of the most common cancers in women worldwide and despite the significant progress in the diagnosis and treatment of breast cancers, there are still more than 40,000 deaths per year [13, 14]. Dysregulation of sex hormones [13–15] is believed to be one of the primary risk factors for breast cancer. A menopause associated decrease in age-specific incidence rates, known as Clemmesen’s hook, is widely observed among females worldwide [16, 17]. The hormonal dysregulation manifests, both clinically and molecularly, as distinct subtypes: triple negative (TN), HER2 positive (HER2+), and ER positive (ER+) [18]. Recently, it has been demonstrated that post-menopausal estrogen metabolism is associated with microbial diversity [19]. Similarly it has been proposed that estrogen conjugation by beta-glucuronidase, may be associated with the microbiota observed to be in dysbiosis in women with a history of breast cancer [20].

The objective of the current study is to characterize the breast microbiota and investigate whether the microbial composition is associated with host expression profiles. This objective was accomplished by utilizing RNA sequencing data from The Cancer Genome Atlas (TCGA), the largest sequencing cohort currently available for breast cancer [18]. We documented the most prevalent species observed among the breast tissues, and gathered preliminary evidence of microbial compositional shifts among the disease subtypes. This was further supported, by 16S-rRNA gene sequencing data obtained from fresh frozen samples that were obtained from the TCGA subjects submitted by the Mayo Clinic. Traditionally, bioinformatic methods such as PICRUSt are implemented to infer the abundance of gene families, which may in turn influence host’s expression response [21]. In contrast, we utilized two lines of evidence- microbial read evidence and host transcriptional expression—from the same tissue and sequencing platform, to perform association analysis. The implementation of a single sequencing platform reduces confounding factors of tissue heterogeneity, molecule extraction/purification, and protocol handling differences. We believe this is the first study to examine both the microbial presence and host expression from the same tissue/sample preparation.

## Materials and methods

### RNA sequencing data

#### Clinical filtering

Clinical characteristics of the breast cancer samples were obtained from the TCGA data portal [22] (November 11, 2013, version). Samples were removed from consideration if they possessed any of the following characteristics: male gender, metastatic samples, history of prior breast cancer disease, recipients of neoadjuvant therapy, and/or samples lacking (documented) interrogation of HER2 amplification status. The fastq sequence files for the remaining 804 breast tumor and non-cancerous breast tumor, were obtained from the Cancer Genomics Hub (CGHuB) data repository[23].

#### Host aligned reads

We aligned RNA-Seq fastq files using TopHat (v1.3.3), mapped reads were used to obtain gene expression counts using HTSeq (v0.5.3p3) [24, 25]. Host gene expression counts were normalized with conditional quantile normalization to account for potential GC, and/or gene length biases [26]. Subtype specific host expression cohorts were inspected for outliers using calibration stress measures [27] and principal component analysis.

#### Non-host aligned reads

To characterize the breast microbiota, the reads unaligned to the host genome were aligned by Kraken to its bacterial genome (03–2014 build) and bacterial 16S ribosomal databases (06–2014 build) [28]. Subsequent to TopHat alignment unmapped BAM files, of the 804 tumor and non-cancerous adjacent (NCA) samples, were converted to FASTQ reads that were quality-trimmed with fastq_quality_trimmer (FASTX Toolkit v0.0.13.2). Read lengths less than 40 nucleotides and a minimum PHRED quality score of less than 3 were removed and the remainder were realigned with Bowtie 2 to eliminate any additional human reads. The left over unmapped reads were then aligned to bacterial genomes and bacterial 16S ribosomal genes using Kraken (v0.10.5-alpha) [28]. Concordance of sample retention, of the parallel processed TCGA samples, further pruned the study cohort to a final cohort consisting of 668 tumor samples and 72 NCA (NCA) reference samples.

### 16S ribosomal sequencing validation

Frozen breast tissue blocks demonstrating greater than 60% tumor composition were selected from six ER+ patients from Mayo Clinic that were submitted to TCGA. Frozen sections (15–20 at 4 μm) were cut from each block and stored at -80°C prior to DNA extraction. The MoBio PowerSoil® DNA Isolation Kit (PN 12888 Mo Bio Laboratories, Inc. Carlsbad, CA) were used for DNA extraction according to the manufacturer’s protocol. The DNA concentrations were measured by Qubit dsDNA HS Assay Kit (PN Q32854 Thermo Fisher Scientific Inc., Waltham, MA) and samples with sufficient DNA were enriched for microbial DNA using the NEBNext® Microbiome DNA Enrichment Kit (PN E2612L, New England Biolabs, Ipswich, MA). The V3-V5 region of the 16S-rRNA gene were amplified with a two-step PCR protocol, and then Illumina flow cell adaptors containing indices were incorporated [29]. Sequence reads (both pairs) were filtered for quality using PHRED quality scores Q3, and 3’ reads with average sliding window (size = 4) score of Q15, using Trimmomatic v 0.22 [30]. Paired reads with that were at least 80% of the original read length nucleotides were aligned with the IM-TORNADO 16S analysis pipeline[31, 32] using the Greengenes taxonomy (Greengenes99 database version 12.10) [33]. Additional details are provided in S1 File.

### Statistical analysis and visualization

#### Batch analysis

To account for dispersion and sparsity, microbial reads were normalized with the metagenomeSeq package [34]. We used the Bray-Curtis dissimilarity measure, a count-based measure to compare between two sampling sites, traditionally employed in ecological studies [35–37]. To identify the presence of handling/processing differences, centroid clustering was performed using the t-SNE dimensionality reduction on the Bray-Curtis dissimilarities [36, 38]. The NbClust package was then employed to determine the optimal number of clusters [39]. This package evaluated the validity of k (2:10) clusters, as scored by 26 indices. The 26 (default) of 30 potential indices were chosen as they are the least computationally expensive. Cluster validity was gaged by the average silhouette width, where a higher average silhouette width indicates high sample similarity (tightness) and substantial cluster separation[40, 41]. Clustering results were confirmed using DESeq2 normalized data and non-negative matrix factorization, see S2 File.

#### Statistical analysis

Concordances of 327 species were observed among the NCA tissues and the tumor samples, and consistent across the observed batch effect. The batch influence was adjusted for with the sva package, using the zero-inflated Gaussian normalized data [34, 42]. Differential abundance analysis of adjusted microbial read evidence was performed with limma for the most abundant operational taxonomic units (OTUs) [43]. Host differential gene expression analysis (human) was performed with edgeR, normalized to a negative binomial distribution to account for read dispersion [44]. Spearman’s correlation was performed at the univariate level, among the OTU’s and genes. Fisher’s exact test was performed for gene set enrichment analysis, using the 50 hallmark pathways [45, 46]. Additionally, mutual information was assessed, with the BUS package [47], among the normalized microbiome data and the 50 hallmark pathways.

#### Visualization

Bean plots were prepared with the Beanplot R package [48] and hive plots with HiveR [49]. Cladograms were produced with GraPhlAn software [50]. Parallel coordinates and parallel sets plots were generated with epade [51].

## Results

### Microbiota identification

The unaligned human genome reads obtained from 668 tumor and 72 NCA tissues were attributed to 1,634 microbial OTUs using dual alignment strategy, which was chosen to minimize contaminants [12, 52, 53]. We further applied a set of filters to remove rare microbial OTUs, those presented in less than 25% of our study cohort, and low abundant microbial OTUs with less than nine maximum reads across the samples. The elimination of OTUs occurring in less than 25% of the samples had little effect on the overall OTU counts of each sample, with 99.2% (657 samples) removing less than 5% of their overall read counts. An ER positive sample (TCGA-BH-A0BR) demonstrated the highest eliminated read counts (10.94%), see S1 Table. As microbial OTUs could be tissue specific, the tumor and NCA samples were grouped and processed in parallel. This left 662 OTUs observed among the tumor samples; 639 OTUs observed among the NCA tissues and with 589 OTUs observed concordantly in both tumor and NCA tissues (S1 Fig). We performed Kmeans clustering analysis using the reduced dimensions from t-distributed stochastic neighbor embedding (t-SNE). The t-SNE mappings were derived from the Bray-Curtis dissimilarity index. We observed that both tumor and NCA cohorts demonstrated the existence of a batch processing effect indicated by the TCGA plate id [54](S1 Fig).

**Fig 1:**
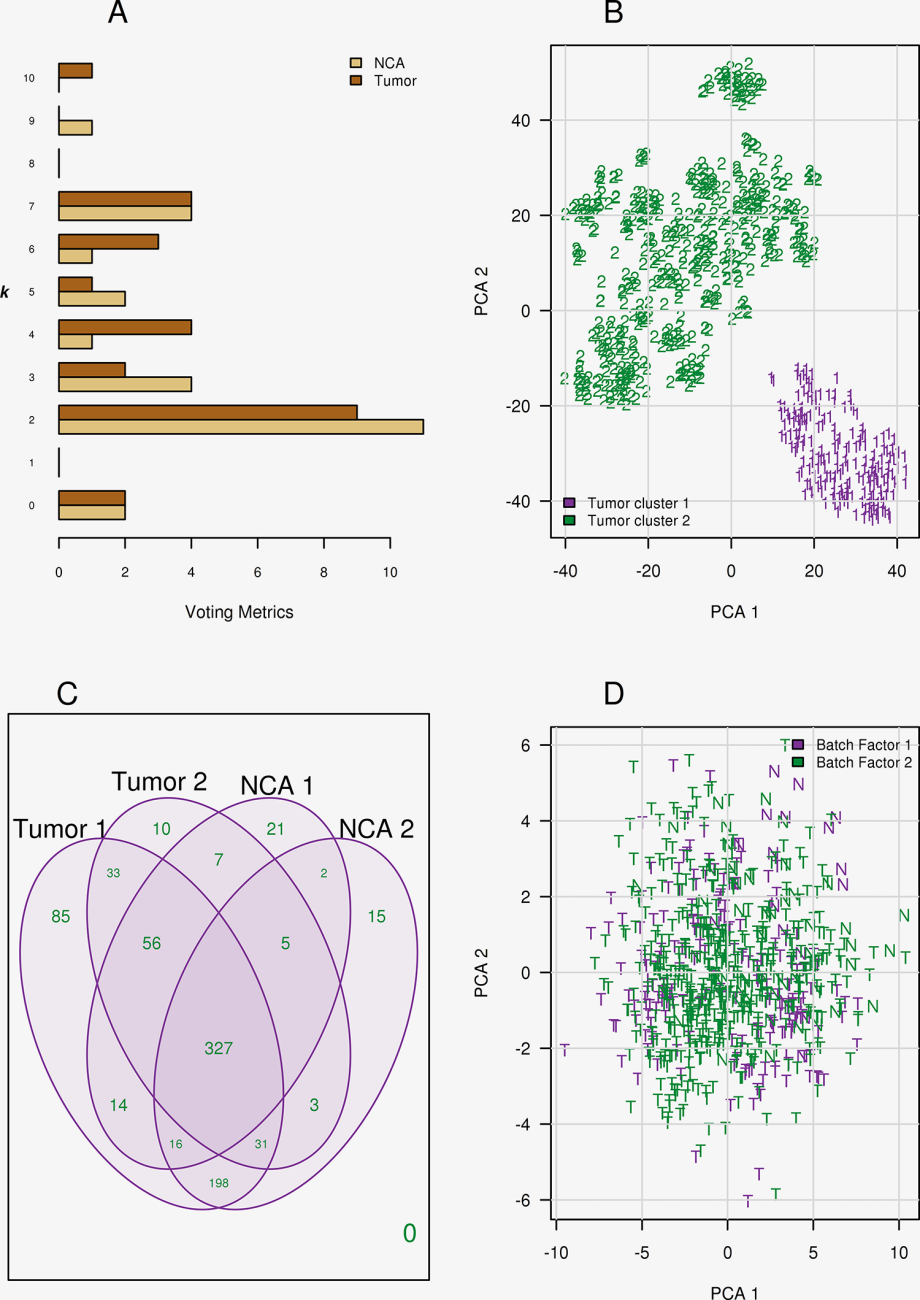
Two distinct microbiota are present in breast tissue samples, both tumor (*n* = 668) and NCA (*n* = 72). (A) Optimal *k*-cluster selection analysis demonstrating a majority decision among the 26 metrics for 2 clusters was concordant for both tissue cohorts identifying a systemic handling bias. (B) The t-SNE projections for the 668 tumors samples, demonstrating tissues demonstrating separation and cohesion among the batches. (C) Filtering was re-applied among the tissue samples and per processing differences. Venn diagram demonstrates a core 327 bacterial OTU’s was observed among all samples. (D) Abundance differences were observed for 48 OTU’s. A PCA plot of these significant OTU’s is presented demonstrating that the batch differences had been accounted for, NCA tissues indicated with an ‘N’ and tumor samples indicated with a ‘T’.

Two clusters were identified for tumor (cluster 1 = 197 and cluster 2 = 471 samples), and NCA cohorts (cluster 1 = 54 and cluster 2 = 18 samples), Fig 1A. A PCA plot is provided in Fig 1B, demonstrating the sample separation created by the batch effect. The filtering describe above was applied to each of the clusters. The tumor cluster 1 (25%) and NCA cluster 2 (25%) demonstrated a concordant set of 198 OTU’s (S2 Table) suggesting the same processing factor was influencing the samples, Fig 1C. The concordant set of 327 OTU’s that were observed among the four clusters was normalized and scaled, S2 Table.

Pairwise differential abundance analysis of the 327 OTU’s was performed among the four tissue groups (NCA, HER2+, ER+, and triple negative breast cancer), S3 Table. Benjamini and Hochberg correction was applied to address multiple testing error and 48 OTU’s were observed to be significant (alpha = 0.05) across the pairwise comparisons. A PCA plot of these final 48 OTU’s is provided in Fig 1D, demonstrating that the batch factor was accounted for among the normalization and scaling process.

### Phyla comparison to Canadian/Irish breast microbiota data

**Fig 2:**
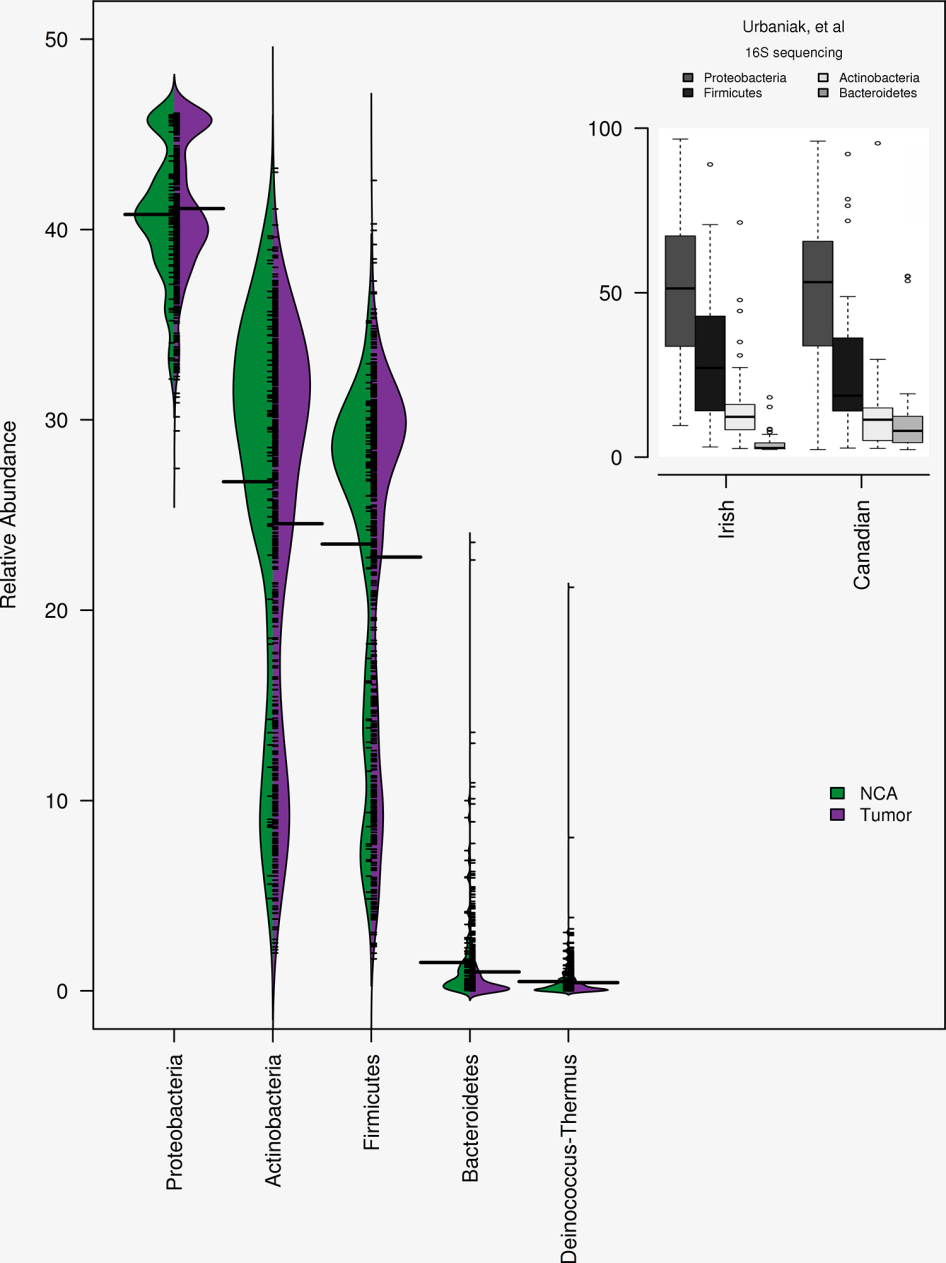
Phylum compositions of the tumor and NCA tissues. The most prevalent phylum in the breast microbiota is *Proteobacteria*, followed by *Actinobacteria* and *Firmicutes*. These observations were consistent among each of the tissue types (tumor shaded in purple NCA shaded in green). These distributions are consistent with the observations by Urbaniak, *et al*, see insert.

Among 740 TCGA RNA Sequencing tumor samples, nearly half of the identified (48.0%, 157 of 327) reads were derived from *Proteobacteria*. The next most prevalent phyla were *Actinobacteria* (26.3%, 86) and *Firmicutes* (16.2%, 53). The remaining 31 OTUs (9.5%) were from miscellaneous phyla. Moreover, the phylum level composition was consistent among the tumor and NCA tissues among the identified clusters (Fig 2). We present the phylum-level distributions as bean plots, in Fig 2, with the tumors (the distribution densities on the right in purple) and the NCA (the distibution densities on the left in green) clusters. These findings were consistent with Urbaniak, *et al*, shown in the upper right inset in Fig 2.

**Fig 3:**
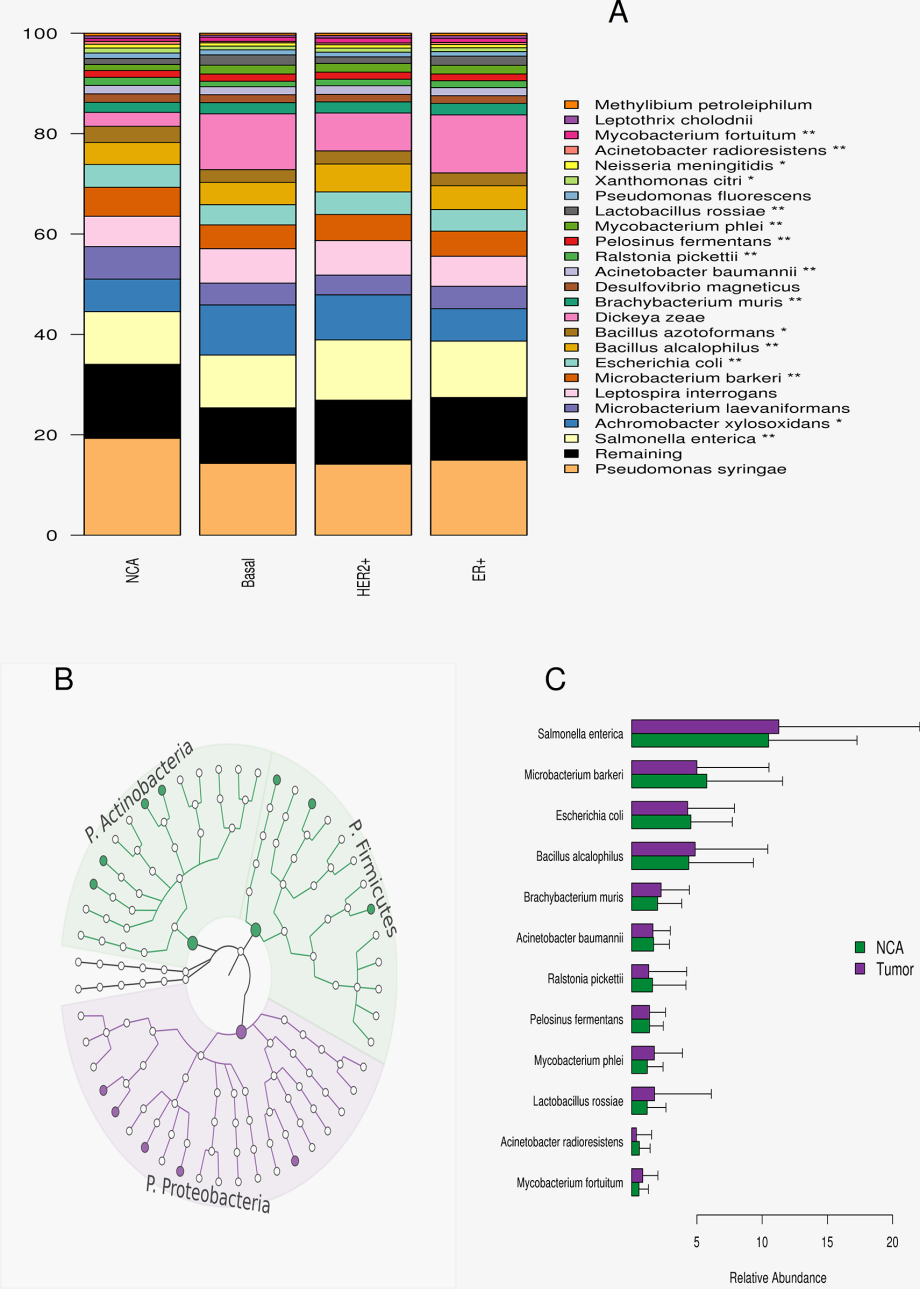
OTU’s significantly different among the breast cancer populations. (A) Twenty-four species were observed to have average abundance between 0.5% - 19.3% among the NCA samples. These species are presented as a barplot summary averaged among each subtype. (B) A cladogram of the 48 differentially abundant species, 12 of the 24 prevelent species (average relative abundance greater than 0.5%) were observed to be signicant after Benjamini and Hochberg correction for mutiple testing error. (C) The average relative abundance and standard deviation of the 12 species differing in abundance depicted in the barplots, with the NCA tissue cohort’s averages in green and tumor cohort averages in purple.

We observed 24 of 327 species with an average relative abundance ranging from 0.5–19.3% in the NCA cohort. However, these 24 species account for 85.64% of the overall breast tissue microbiota, across the three breast tumor subtypes as well as the NCA tissue; as presented in Fig 3A. The remainder of the less prevalent microbiota, on average, comprises: 14.7% of the NCA microbiota, 11.1% of the triple negative microbiota, 12.8% of the HER2+ microbiota and 12.5% of the ER+ microbiota.

#### Significant OTU’s

Differential abundance analysis identified 48 OTU’s to be significant after correcting for multiple testing errors using Benjamini and Hochberg correction to control the false discovery rate. Nine species were concordantly observed to have absolute abundance differences (fold change) greater than 2 among the breast cancer subtypes, in contrast to the NCA tissues. These 48 species are presented as a cladogram in Fig 3B; the nine species observed to be significantly altered from NCA tissues are presented as shaded circles. The relative abundances of these nine species are also shown in Fig 3C, contrasting the tumor cohort distributions and NCA microbial distributions.

We observed that the prominence of differentially abundant species were comprised from the less prevalent OTU’s, as only 12 of the 48 (25%) comprised the 24 more prevalent species. Similarly, the majority (6) of the nine OTU’s significantly altered from NCA tissues were also comprised from the less prevalent OTU’s. *Mycobacterium fortuitum* and *Mycobacterium phlei*, both known to infect humans were two of the prevalent species observed to be differentially abundant in the tumor samples (Fig 3C). Additionally, we observed an increased presence of *Actinobacteria* (13 of 48 significant OTU’s) in the NCA tissue samples; while *Proteobacteria* (20 of the 48 significant OTU’s) demonstrated increased presence among the tumor tissues, see S2 Fig.

### Microbial association with breast cancer

**Fig 4:**
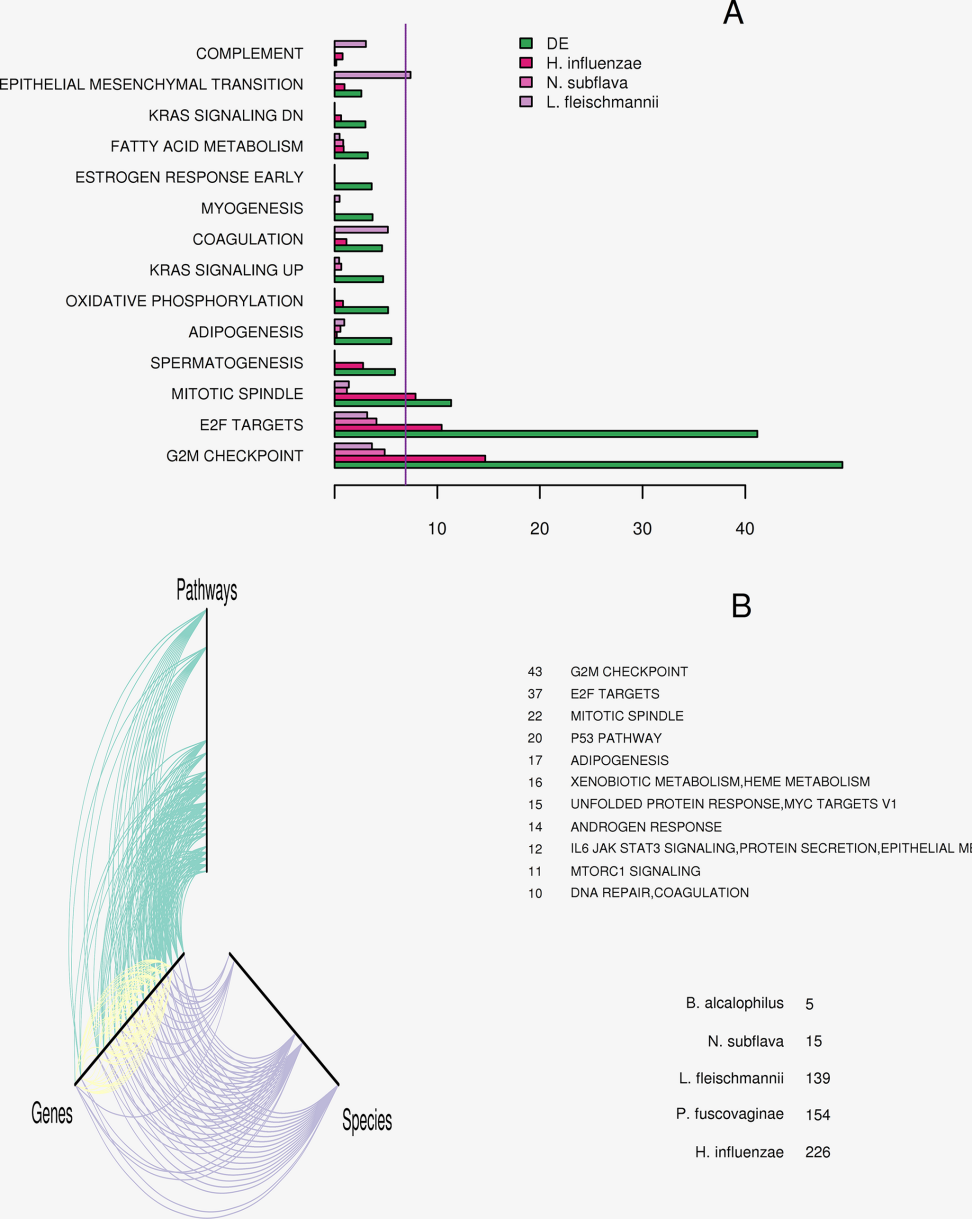
Microbial association with breast cancer. A) Fourteen hallmark pathways which were observed to significantly enriched with differentially expressed genes (in green), versus the universe set of 16,363 genes. Three organisms (*H*. *influenza*, *N*. *Subflava*, *and L*. *fleischmanni*) demonstrated correlation to a sufficient set of genes for geneset enrichment analysis: 229, 30, and 58, genes respectively. *H*. *influenze* shared enrichment in three of those pathways. Those three pathways (G2M checkpoint, E2F targets, and mitotic spindle), remain enriched, after applying Bonferroni correction (the vertical purple dashed line). *L*, *Fleischmannii* was also enriched in gene correlations among the epithelial mesenchymal transition pathway, after Bonferroni correction for multiple testing. B) To reduce any bias from differential expression selection the genes in the 50 hallmark pathways were additionally analyzed for mutual information content with the microbial compositional data. A hive plot depicting the network of the 50 pathways and their genes (green), known gene-gene (protein-protein) interactions (yellow), and OTU’s-gene associations (purple) is presented. The connectivity of the pathway and OTU’s nodes are presented with the text on the right. We again observe the same 3 networks demonstrated the most connectivity and that *H*. *influenza* as the predominant microbial association.

We investigated whether the microbial composition was associated with alterations in the host expression profiles. EdgeR analysis was performed between the NCA tissue and the breast cancer subtype tissue using host gene expression data. We identified 683 common genes demonstrating logFC greater than two and a Bonferroni corrected p value less than 0.05. We conducted a correlation analysis using the 683 genes and the 48 OTU’s. Genes demonstrating a Spearman’s correlation (absolute) greater than 0.25 with the OTU’s, (S5 Table), were kept for geneset enrichment analysis for the 50 hallmark pathways. Three organisms (*H*. *influenza*, *N*. *Subflava*, *and L fleischmannii* were interrogated since they demonstrated sufficient gene correlations for enrichment analysis, with 229, 30, and 58 genes respectively. Fisher’s exact test compared each pathway to the reference set of 683 differentially expressed genes. In addition, the gene set enrichment of the pathways for these 683 differentially expressed versus the entire reference set of 16,383 expressed genes, was evaluated as a benchmark for design bias. We observed 14 pathways to be enriched in the 683 differentially expressed genes. Two pathways (G2M checkpoint and E2F targets), were enriched in all four comparisons, while mitotic spindle was enriched in all but *N*. *Subflava*. However these pathways do not survive any adjustment for multiple testing errors, except in the overall differentially expressed and correlated to *H*. *influenza*’s composition, where they survive the most stringent Bonferroni correction. Additionally, *L*. *fleischmannii* remained associated with epithelial mesenchymal transition, after adjusting for the family-wise error rate with the Bonferroni adjustment. The enrichment results are presented as the negative natural log of the p values, in the Fig 4A. We note that the pathway associations with *H*. *influenza* may follow the general bias of selecting the differentially expressed genes.

We conducted a second association analysis in an effort to reduce the impact of expression bias, by assessing all expressed genes in the 50 hallmark pathways. Mutual information analysis was performed between the microbiota compositional data and expression profiles. The mutual information is empirically estimated based upon the entropies of each variable’s distribution and the combined variables distribution. A hive plot is presented in the bottom of Fig 4B, depicting the observed associations among the gene expression, microbiota, and the hallmark pathways. A hive plot collapses a network diagram into 3 axes, where each node represents variables sharing the same connectivity. The more connections (associations) observed the further away from the center origin (x = 0, y = 0, and z = 0). We have provided text detailing the observed association patterns. We observe that same 3 pathways remain the most enriched in genes sharing information with the microbiota, with G2M possessing 43 uniquely associated genes. Similarly *H*. *influenza* remained the most associated microorganism, with 226 associated genes. While *L*. *fleischmannii* (139) and epithelial to mesenchymal transitions (12) remained prominently connected features.

### 16S-rRNA gene sequencing for confirmation

To confirm the presence of microbiota in the TCGA breast samples, for whom the fresh frozen tissue was available, we generated standard 16S-rRNA gene sequencing in a subset of TCGA samples (n = 6) contributed by the Mayo Clinic [18]. In order to control for contamination we included negative controls of the DNA extraction and PCR amplification solutions. Minimal artefactual read evidence was observed for *G*e*obacillus spp*, as a result of the spiked in DNA enrichment step (14 and 10 reads) and therefore subtracted from our sample cohort (S2 Table and S1 File). The fresh frozen samples were otherwise observed to be predominated by the *Proteobacteria* phylum, consistent with the previously published study of 16S-rRNA gene sequencing with 81 women [10, 55] (S2 Table). Similarly, multiple species were concordantly observed for *Streptococus*, *Lactobacillus*, *and Acinetobacter* [10, 55] (Table 1), while we observed *Gluconacetobacter* as the most prevalent OTU in terms of high read evidence and consistently across samples.

**Table 1:** Concordantly observed OTU’s. The *Proteobacteria* and *Firmicutes* species which were concordantly observed in the 16S-RNA gene and mRNA sequencing data. Additional 16S read data were observed, yet remained uncharacterized at the species level, which could coincide with mRNA observations.

Phyla	Genus	16S and mRNA Confirmed (Species level)	16S Observed (Genus level)
*Firmicutes*	*Lactobacillus*	*Lactobacillus iners*, *Lactobacillus helveticus*	*Lactococcus* unclassified
	*Streptococcus*	*Streptococcus infantis*	*Streptococcus alactolyticus*
	*Propionibacterium*	*Propionibacterium acnes*	* *
	*Staphylococcus*	*Staphylococcus epidermidis*	* *
*Proteobacteria*	*Pseudomonas*	*Pseudomonas veronii*, *Pseudomonas stutzeri*	* *
	*Haemophilus*	*Haemophilus parainfluenzae*	* *
	*Acinetobacter*	*Acinetobacter johnsonii*, *Acinetobacter lwoffii*	* *
	*Sphingomonas*	* *	*Sphingomonas* unclassified
	*Agrobacterium*	* *	*Agrobacterium* unclassified

## Discussion

We utilized the unaligned RNA sequence reads available from the TCGA, a multi-institution study and the largest known sequencing cohort available for breast cancer samples. A dual alignment of high quality RNA reads was implemented as false identification was of utmost concern in microbiota identification [52, 53]. Stringent filtering on data sparsity was employed to ensure the identification of the most prevalent OTUs. Additionally, prior to our analysis we removed low quality and/or low RNA yielding samples, as these samples are more susceptible to contaminant amplification [53]. A prominent batch effect was observed accumulating in spurious read evidence for 198 species (Fig 1B and 1C, S1 Table). The ComBat algorithm demonstrated superior performance in adjusting the compositional data normalized with a zero inflated Gaussian model over negative binomial models (data not shown).

Fresh frozen samples, which were from the analogous TCGA patient source, were also assayed with 16S-rRNA gene sequencing (S6 Table). Both datasets confirmed that the most abundant phyla in breast tissues are *Proteobacteria*, *Actinobacteria*, and *Firmicutes* (Fig 2), similar to that reported by smaller 16S-rRNA gene sequencing studies of breast tissue [10, 55]. Comparatively, the mRNA sequencing data appears to be noisier in composition, with over 300 identified species. However, the batch effect which we observed among the TCGA data would likely not have been identified using 16S-rRNA gene sequencing, given the sparsity of the data. While these preparations originate from the same patient, the datasets are derived from distinct laboratory protocols and potentially more importantly different tissue preparations. While the *in silica* processing was different, we were able to concordantly identify ten OTU’s at the species level, while an additional three unclassified OTU’s were also identified (Table 1, S7 Table). While seemingly unimpressive, the 16S-rRNA gene sequencing approach identified only 30 OTU’s, of which only 18 were characterized to named species. The disconnect in tissue preparation and results illustrates the major advantage of implementing the mRNA sequencing approach: the ability to examine two components of the tumor microenvironment using data originating from the same source and *in vitro* processed in the same way.

We identified forty-eight significant OTU’s after Benjamini and Hochberg correction for multiple tests. Nine of which had absolute fold changes greater than two, for each breast cancer subtype compared to NCA tissues (S3 Table). Interestingly, the vast majority (75%, 36 of 48) were found to comprise less prevalent OTU’s, less than 0.5% on an average in the NCA). While, this may be attributed to the tumor-biased nature of the TCGA data, it could also suggest that breast cancer studies with limited sampling power coupled with sparse microbiome data could be inadequate for accurate signal detection. We observed 24 prevalent (greater than 0.5% on average in the NCA) species across the three breast tumor subtypes and the NCA. These 24 species constituted 85.64% of the overall breast tissue microbiota. Twelve of these twenty-one species were observed to be significantly altered after adjusting for multiple testing errors using Benjamini and Hochberg, across the four 4 breast tissue phenotypes (Fig 3A–3C).

We provide confirmatory evidence that *Escherichia coli* as one of the more prevalent species in the breast tissue and is observed in higher abundance within NCA breast tissues [1, 11, 56]. *Escherichia coli* has been reported to have the ability to inflict DNA damage through its production of colibactin, and could lead to genomic instability which triggers oncogenic process [55]. Similar to published results we observed differential abundance among *Cornebacterium*, *Corynebacterium*, and *Bacillus* and the *enterobaceriaceae*: *E*. *coli* and *Salmonella enterica [11]*. The *Firmicutes* were represented by 13 significant species, including two *Lactobaccillus spp*. and five *Streptococcus spp*. Another important manner by which breast microbiota could influence oncogenesis is by enhancing the local exposure of breast tissue to estrogen levels. Previously fecal studies demonstrated positive correlations between the abundance of *Streptococcus* and the presence of β-glucuronidase and/or β-glucosidase enzymes, which cleave the estrogen-glucuronide conjugate and promote recirculation of estrogen [57, 58]. Systemic estrogen levels have been widely recognized to be associated with enhanced breast cancer risk [59] and recent 16S-rRNA gene sequencing experiments reports have associated glucuronidase prevalence in nipple aspirate fluid of breast cancer survivors [20]. The expression profiles for glucosylceramidase beta 2 (GBA2) and the glucuronidase, beta pseudogenes 4 and 9 were positively correlated with *S*. *pyogenes*. We observed abundance increases specifically for *S*. *pyogenes* and *L*. *rossiae* in the tumor samples.

Finally, we interrogated whether any of these OTUs could be associated with host tumor expression profiles. We observed *L*. *fleischmannii* and to a lesser extent *N*. *Subflava* to be correlated with tumor gene expression. *Listeria fleischmannii* was more strongly associated with genes involved in the epithelial to mesenchymal transition (Fig 4A). In the same analysis we observed *H*. *influenza* to be correlated with genes representing pathways fundamental to tumor growth: G2M checkpoint, E2F signaling, and mitotic spindle assembly (Fig 4A and S4 Table). These findings were validated with an unsupervised approach (Fig 4B and S5 Table). *H*. *influenza* is an opportunistic pathogen which has been demonstrated to elicit an inflammatory immune response and promote tumor growth in murine lung cancer models [60, 61]. We observed an increased presence of *H*. *influenza* in the NCA samples, suggesting that the organism may more predominantly reside in the surrounding stromal tissue. Further, *H*. *influenza* has been demonstrated to be susceptible to H_2_O_2_ production, suggesting that the oxidative tumor environment may be prohibitive to *H*. *influenza* penetrance and thereby restricting its presence to the surrounding stroma.

## Conclusions

In this study, we utilized the largest available collection of TCGA breast cancer RNA sequencing data to examine the microbiota residing in breast tissues. We observed abundance shifts in *Proteobacteria* in tumor samples, while shifts in *Actinobacteria* abundance favored NCA tissues. We observed that the significant shift in microbiota typically involved the less prevalent OTU’s, which suggests that under-powered studies are likely to report erroneous results. A semi-supervised approach was implemented to correlate expression profiles with the microbiota compositional data, which revealed *H*. *influenza* to be significantly correlated with genes in the G2M checkpoint, E2F transcription, and mitotic spindle assembly pathways. Similarly, *L*. *fleischmannii* was observed to be associated with genes involved with epithelial to mesenchymal transitioning. Additionally, we report correlations between *S*. *pyogenes* abundance and GUSBP4, GUSBP9, and GPA2 expression levels, which may incriminate the role of *S*.*pyogenes* in the glucuronidation of estrogen and exposing local breast environment to higher estrogen levels. These findings further support the importance of the host tumor expression in light of the microbial presence within the tumor microenvironment.

## Supporting information

S1 File 16S-rRNA sequencing protocols. (PDF)

S2 File Cluster confirmation. Analysis of institutional sampling sources, eliminating Subsequent analysis of tumor data, normalized with DESeq2 and clustered with non-negative matrix factorization.
(PDF)

S3 File 198 potentially spurious OTU’s. (TXT)

S1 Fig Two clusters were present in TCGA breast tissue samples, tumor (*n* = 668) and NCA (*n* = 72). (A) Venn diagram of the bacterial OTU’s observed among the tumors and NCA, demonstrating similar microbial presence. (B) Optimal *k*-cluster selection analysis demonstrating a majority decision among the 26 metrics for 2 clusters was concordant for both tissue cohorts. (C) A PCA plot of NCA tissues demonstrating separation and cohesion among the smaller second cluster of samples. (D) A perspective plot of the tumor samples demonstrating the sharpness and isolation of tumor samples designated as tumor cluster 1. (E) Silhouette plot for the NCA samples demonstrate a significant proportion, (normal cluster 2, 18 of 72, 0.56) present a distinct batch processing factor which needs to be accounted for. (F) Similarly, the silhouette plot among the tumor samples also demonstrates that a significant proportion (tumor cluster 1,197 of 668, 0.4) also need to be accounted for.
(TIF)

S2 Fig Parallel sets of the log fold changes observed among the 48 significant OTU’s, aggregated among the three major phyla. The observed log fold changes were binned for each of the subtype comparison again NCA tissues.
(TIF)

S1 Table Retention of the prevalent operational taxonomic units. Table documenting the percent of read count evidence retained as a result of filtering out taxonomic units low abundant OTU’s (with less than nine maximum reads across samples) and less prevalent (observed in less than 25% of the sampling population.
(TSV)

S2 Table SVA batch adjusted microbiota compositional data. (TSV)

S3 Table Limma analysis of the adjusted microbiota compositional data. (TSV)

S4 Table Spearman correlation of the adjusted microbiota compositional data to RNA Sequencing expression profiles. (CSV)

S5 Table Mutual information of the adjusted microbiota compositional data to RNA Sequencing expression profiles. (TSV)

S6 Table Microbial read evidence from 16S-rRNA sequencing. Operational taxonomic units for 16S-rRNA sequencing analysis of fresh-frozen tissues samples corresponding with Mayo ER+ contributed TCGA breast cancer samples. The identification of *Geobacillus spp*. is resultant from a DNA spike–in for DNA enrichment in the NEBNext amplification.
(TSV)

S7 Table Microbial read evidence of species identified concordantly by the two sequencing technologies. Fresh frozen tissue samples from six Mayo Clinic ER+ samples submitted toTCGA were subsequently analyzed with 16S-rRNA gene sequencing for their microbial content. Read evidence for 30 OTU’s (31 with the identification of *Geobacillus vulcani* amplification material) were identified, with 18 (60.%) characterized to the species level. Ten of the eighteen (55.6%) characterized species were confirmed with the mRNA sequencing data.
(TSV)
