# Supplementary material for: A comprehensive analysis of breast cancer microbiota and host gene expression
Source: PLoS One. 2017 Nov 30;12(11):e0188873. doi: 10.1371/journal.pone.0188873 (PMC5708741; doi:10.1371/journal.pone.0188873)
Supplement: S1 File — (PDF) [file pone.0188873.s001.pdf]

## Samples

Frozen breast tissue blocks were obtained from 7 patients. Blocks with 60% or greater tumor were selected based on pathology review of H&E staining. Normal adjacent tissue from 3 patients was utilized as a control.

## Collection procedure

The cryostat was thoroughly cleaned with using 5 minutes exposure of Oxivir TB (Diversey Care, Racine, WI ) prior to use. The blade and block holder were similarly cleaned after each block was cut to ensure there would not be any cross-over contamination of samples. The cryostat blade was swabbed (PN 551CBX, Copan Diagnostics Inc., Murrieta, CA) before each block was cut to monitor for environmental and cross over contamination. Approximately 15-20, 4um frozen sections were cut from each block into precooled 2.0 mL DNA-free micro tubes (PN 72.694.416, Sarstedt INC., Newton NC). Tissue sections and swabs heads were deposited into precooled 2.0 mL DNA-free micro tubes (PN 72.694.416, Sarstedt Inc. Newton, NC) and stored -80 prior to extraction.

## DNA extraction and enrichment

DNA was extracted using MoBio UltraClean® Tissue &  Cells DNA Isolation Kit (PN 12334 Mo Bio Laboratories, Inc. Carlsbad, CA) according the manufacturer's protocol. The contents of the supplied power bead tubes were transferred to the sample collection tube immediately before extraction. DNA  DNA concentration was measured by Qubit dsDNA HS Assay Kit (PN

Q32854 Thermo Fisher Scientific Inc., Waltham, MA). Tissue samples with sufficient DNA were enriched for microbial DNA using the NEBNext® Microbiome DNA Enrichment Kit (PN E2612L, New England Biolabs, Ipswich, MA) following the manufactures instructions. To insure all samples could be sequenced duplicate PCR reactions were spiked with 30pg of Geobacillus stearothermophilus DNA (PN 12980D-5, ATCC, Manassas, VA)

**Dual indexing 16S PCR**

A two- step PCR protocol was used to amplify the V3-V5 region of the 16S rRNA gene and then add Illumina flow cell adaptors containing indices[1]. Briefly, the primary qPCR reactions were monitored in real time using a Quant Studio 6 Flex real-time PCR system (Thermo Fisher Scientific Inc., Waltham, MA). 6 uL reactions were assembled using the following volumetric percentages: 50% DNA, 20% 5X KAPA PCR Buffer, 16.5% PCR grade water, 5% DMSO (Sigma-Aldrich, Saint Louis, MO.), 3% KAPA dNTPs (10uM), 2% ROX (25uM, Thermo Fisher Scientific Inc., Waltham, MA), 2% KAPA HIFI polymerase, 0.5% SYBR Green (200X, Thermo Fisher Scientific Inc., Waltham, MA), 0.5% forward primer (100uM,) and 0.5% reverse primer (100uM). KAPA reagents were supplied as a part of the KAPA HiFi Hot Start kit (PN KK2502, KAPA Biosystems, Woburn, MA). Sample were amplified with the following conditions: 95 ˚C for 5 minutes, 25 cycles of: 98 ˚C for 20 seconds, 55˚C for 19 seconds, and 72˚C for 60 seconds, and a final 72˚C extension for 5 minutes.  V3_357F and V5_926R primers[2] modified with Nextera adaptors were developed in collaboration with the University of Minnesota Genomic Center in Minneapolis, MN.

*V3_341F_Nextera*:

TCGTCGGCAGCGTCAGATGTGTATAAGAGACAGCCTACGGGAGGCAGCAG

*V5_926R_Nextera:*

GTCTCGTGGGCTCGGAGATGTGTATAAGAGACAGCCGTCAATTCMTTTRAGT

Primary PCR products were diluted 1:100 in PCR grade water for secondary PCR reactions. 10uL reactions were assembled using the following volumetric percentages: 50% DNA, 20% 5X KAPA PCR Buffer, 5% DMSO (Sigma-Aldrich, Saint Louis, MO.), 3% KAPA dNTPs (10uM), 2% KAPA HIFI polymerase, 10% forward primer (5uM,) and 10% reverse primer (5uM). KAPA reagents were supplied as a part of the KAPA HiFi Hot Start kit (PN KK2502, KAPA Biosystems, Woburn, MA). PCR cycling conditions were 95 ˚C for 5 minutes, 10 cycles of: 98 ˚C for 20 seconds, 55˚C for 15 seconds, and 72˚C for 60 seconds, and a final 72˚C extension for 5 minutes. The following indexing primer design was utilized[1] (X indicates the position of the indices).

*Forward i5 primer*:

AATGATACGGCGACCACCGAGATCTACACXXXXXXXXTCGTCGGCAGCGTC

*Reverse i7 primer*:

CAAGCAGAAGACGGCATACGAGATXXXXXXXXGTCTCGTGGGCTCGG

**Normalization and pooling of 16S libraries**

PCR products were diluted to 20uL with PCR grade water and cleaned up using 1.0X AMPureAP beads (Beckman Coulter, Brea, CA), vacuum-dried, reconstituted in 12uL of PCR grade water, quantified using a Quant-It dsDNA HS assay kit (Thermo Fisher Scientific Inc., Waltham, MA), normalized and pooled. The sequencing pool was concentrated, cleaned up using 1.8X AMPureAP beads (Beckman Coulter, Brea, CA) quantified using a Quant-It dsDNA HS assay kit (Thermo Fisher Scientific Inc., Waltham, MA).Sequence pool was assessed for purity and the presence of 725bp peak (± 20%) using a 2200 TapeStation system and D1000 Screen tape/ reagents (Agilent Technologies, Santa Clara, CA).

**Sequencing**

The 16S amplicon pools are quantified using the KAPA SYBR FAST qPCR kit (KAPA Biosystems, Woburn, MA), diluted to 2nM, denatured with an equal volume of 0.2N NaOH, diluted to 6pM with Illumina HT1 buffer, spiked with 30% PhiX, heat denatured at 96˚C for 5 minutes and sequenced using the MiSeq 600 cycle v3 kit (Illumina, San Diego, CA) and MCS v2.6.1.

## Pipeline for processing of 16S data

Pre-processed sequence files are then subject to quality filtering using Trimmomatic version 0.22[3], with a hard cutoff of PHRED score Q3 for 5′ and 3′ ends of the reads (parameters LEADING:3 and TRAILING:3), trimming of the 3′ end with a moving average score of Q15, with a window size of 4 bases (parameter SLIDINGWINDOW:4:15), and removing any remaining reads shorter than 80% of the original read length (parameter MINLEN: 240 for reads of 300 bp long). Finally reads with any ambiguous base calls or with homo-polymers longer than 10 bases longs are discarded using Mothur[4]. Only the read pairs that survived the quality filter were processed further. Any surviving reads that were unpaired (that is, they lost their matching pair due to low quality) were discarded. Surviving read pairs were then grouped into two files, one each for "read 1" and "read 2" sequences. Reads were also de-replicated, consolidating identical reads to avoid redundant processing[5].

## Taxonomy assignment

To prepare the reads for this step, we took reads from the previous step just before the stitching procedure, remove the gaps and then stitched them with a pad sequence of "N" bases. The specific scripts used for these file manipulations are publically available as part of the IM-TORNADO 16S rRNA analysis pipeline (http://sourceforge.net/projects/ imtornado/). Since by default most Bayesian classifiers use 8-mers to perform the classification, we used "NNNNNNNN" as the padding. The stitched reads were then classified using the Greengenes taxonomy (Greengenes99 database version 12.10) as the reference[6] using the following Mothur "classify.seqs" command with "iter = 1000".

## Clustering, representative sequences, and chimera removal

Paired-end reads were concatenated directly with no padding and de-replicated. OTU representatives were selected and used to generate a reference set for clustering using USEARCH command “usearch7.0.1090_i86linux32”[7]. Clustering width was set at 97% (−otu_radius_pct 3.0).


1. Gohl DM, Vangay P, Garbe J, et al. Systematic improvement of amplicon marker gene methods for increased accuracy in microbiome studies. *Nat Biotechnol.* 2016.
2. Yu Z, Morrison M. Comparisons of different hypervariable regions of rrs genes for use in fingerprinting of microbial communities by PCR-denaturing gradient gel electrophoresis. *Appl Environ Microbiol.* 2004;70(8):4800-4806.
3. Bolger AM, Lohse M, Usadel B. Trimmomatic: a flexible trimmer for Illumina sequence data. *Bioinformatics.* 2014;30(15):2114-2120.
4. Schloss PD, Westcott SL, Ryabin T, et al. Introducing mothur: open-source, platform-independent, community-supported software for describing and comparing microbial communities. *Appl Environ Microbiol.* 2009;75(23):7537-7541.
5. Kang SS, Jeraldo PR, Kurti A, et al. Diet and exercise orthogonally alter the gut microbiome and reveal independent associations with anxiety and cognition. *Mol Neurodegener.* 2014;9:36.
6. DeSantis TZ, Hugenholtz P, Larsen N, et al. Greengenes, a chimera-checked 16S rRNA gene database and workbench compatible with ARB. *Appl Environ Microbiol.* 2006;72(7):5069-5072.
7. Edgar RC. UPARSE: highly accurate OTU sequences from microbial amplicon reads. *Nat Methods.* 2013;10(10):996-998.
