# Supplementary material for: A comprehensive analysis of breast cancer microbiota and host gene expression
Source: PLoS One. 2017 Nov 30;12(11):e0188873. doi: 10.1371/journal.pone.0188873 (PMC5708741; doi:10.1371/journal.pone.0188873)
Supplement: S2 File — Analysis of institutional sampling sources, eliminating Subsequent analysis of tumor data, normalized with DESeq2 and clustered with non-negative matrix factorization. (PDF) [file pone.0188873.s002.pdf]

The Breast Cancer Microbiome and Host Biology
short: GLBIO Review Comments

Kevin J. Thompson[1,2], James N. Ingle[3], Xiaojia Tang[1,2], Nicholas Chia[4,2], Jeraldo R. Patricio[4,2], Marina R. Walther-Antonio[4,2], Karunya K. Kandimalla[5], Stephen Johnson[1,2], Janet Z. Yao[2], Sean C. Harrington[2], Vera J. Suman[1], Liewei Wang[6], Richard L. Weinshilboum[6], Judy C. Boughey[4], Jean-Pierre Kocher[1,2], Heidi Nelson[4], Mathew P. Goetz[3], Krishna R. Kalari[1,2]

[1]Department of Health Sciences Research, Mayo Clinic, Rochester, MN;
[2]Department of Center for Individualized Medicine, Mayo Clinic, Rochester, MN;
[3]Department of Oncology, Mayo Clinic, Rochester, MN;
[4]Department of Surgery, Mayo Clinic, Rochester, MN;
[5]Department of Pharmaceutics, University of Minnesota, Minneapolis, MN;
[6]Department of Molecular Pharmacology & Experimental Therapeutics, Mayo Clinic, Rochester, MN;

July 28, 2017

1 Instituional Sampling Source

We investigated whether the microbial differences were arising from the specific Institutional Collecting Agencies and did not observe any systemic biases.

Table 1: Subtypes per Institutional Source

	Basal	HER2	Luminal
A1	0	1	8
A2	17	11	55
A7	3	0	12
A8	4	19	38
AC	0	6	11
AN	5	12	17
AO	8	2	25
AQ	1	3	1
AR	9	7	37
B6	0	0	1
BH	17	18	81
C8	6	17	10
D8	12	11	40
E2	12	12	48
E9	0	10	9
EW	8	4	19
GI	1	0	0
GM	5	1	11
JL	0	2	0
LL	1	0	0

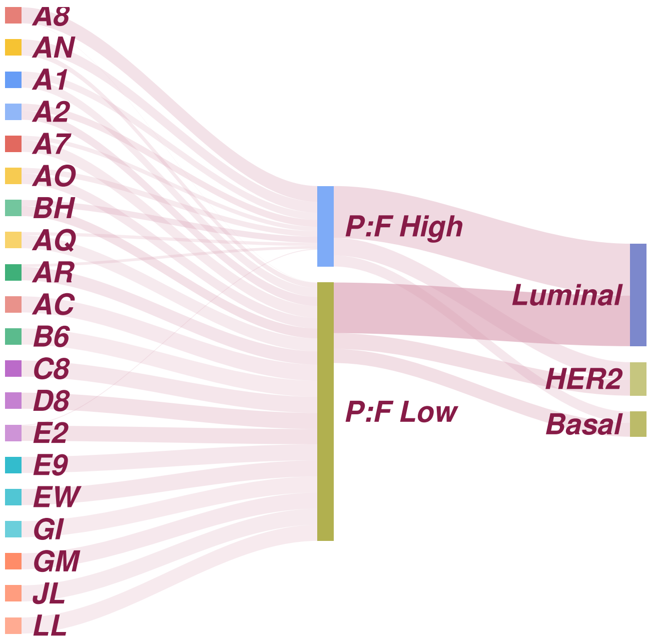

src	target	counts
P:F High	Basal	27
P:F High	HER2	43
P:F High	Luminal	127
P:F Low	Basal	82
P:F Low	HER2	93
P:F Low	Luminal	296

src	target	counts
A1	P:F High	4
A1	P:F Low	5
A2	P:F High	36
A2	P:F Low	47
A7	P:F High	4
A7	P:F Low	11
A8	P:F High	60
A8	P:F Low	1
AC	P:F High	0
AC	P:F Low	17
AN	P:F High	23
AN	P:F Low	11
AO	P:F High	12

Data: various • Chart ID: Tabular • googleVis-0.6.2
R version 3.2.0 (2015-04-16) • Google Terms of Use • Data Policy: See individual charts

2 Clustering Space influence

We investigated whether the use of Bray-Curtis dissimilarity space was influencing our clustering results. We present conordant results with hierarchical clustering in Euclidean space

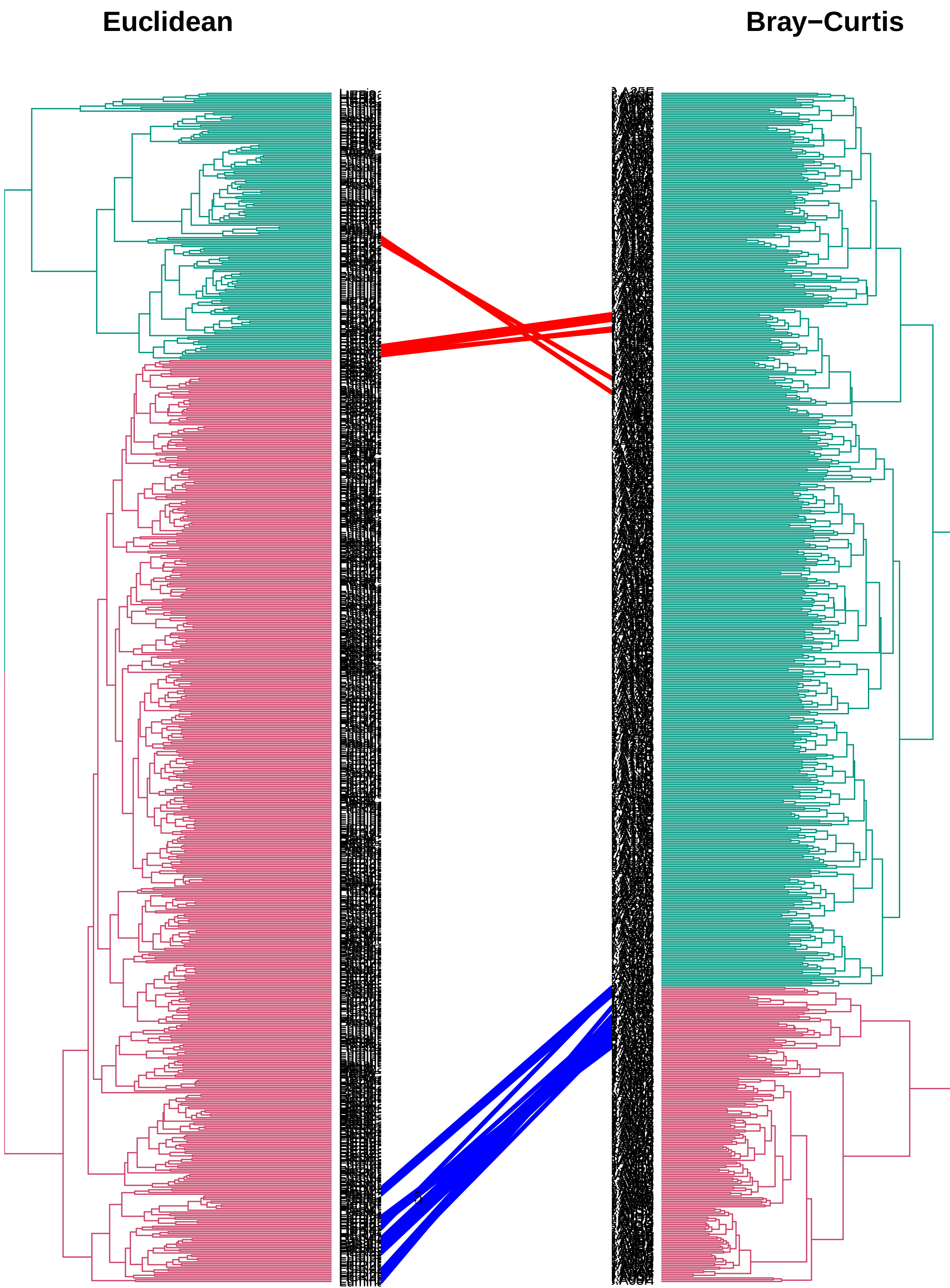
Euclidean
Bray−Curtis

3 Independent normalization and Clustering

We evaluated the ER+ tumor data, from the University of Pittsburgh to remove several confound factors. This institution provided the largest amount of samples, particularly ER+ samples. Normalization was performed with DESeq2 and noon-negative matrix factorizatio was preformed. We demonstrate that we found similar clustering results.

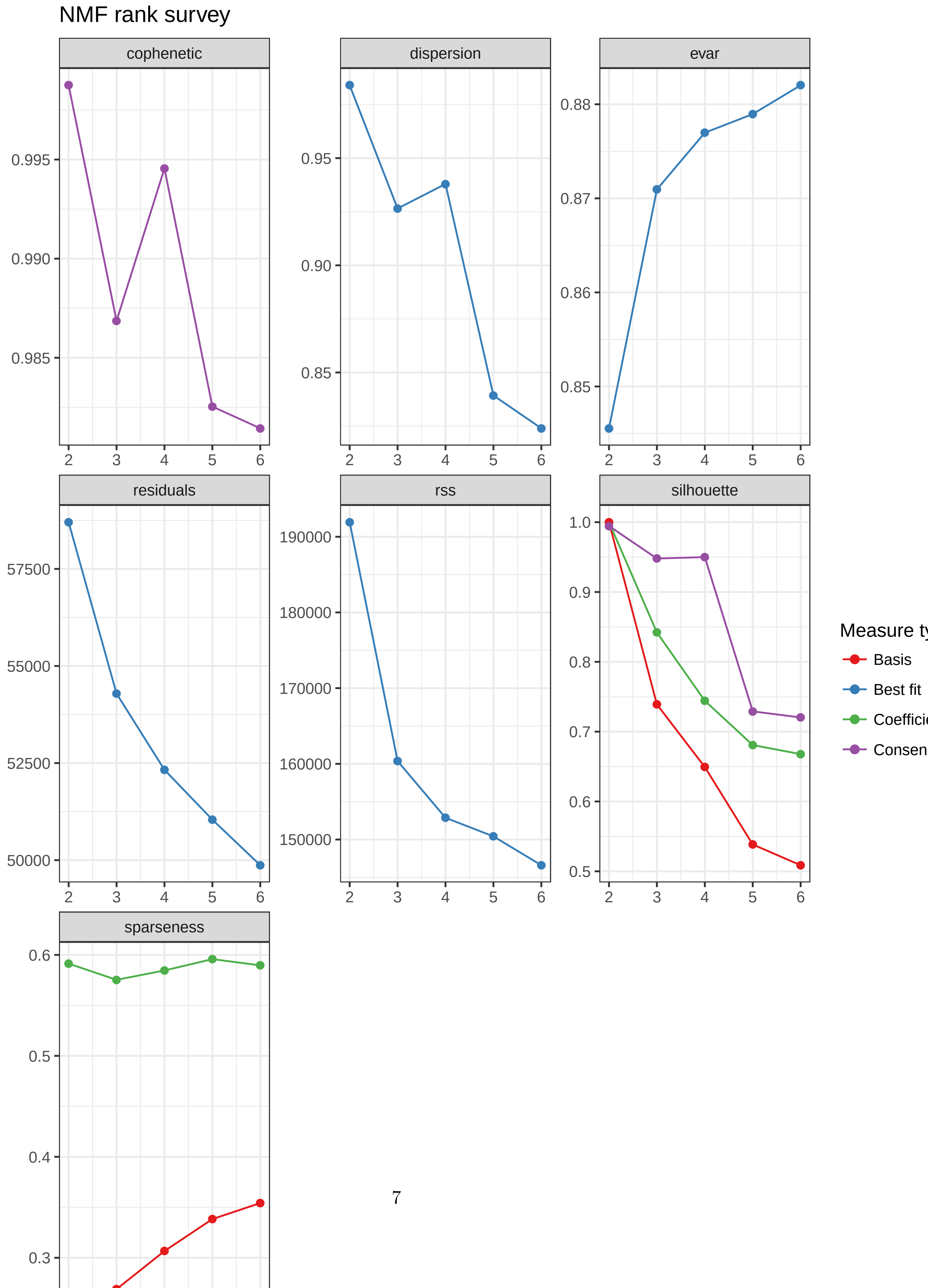
NMF rank survey
cophenetic
dispersion
evar
residuals
rss
silhouette
sparseness
Measure ty
Basis
Best fit
Coeffici
Consen

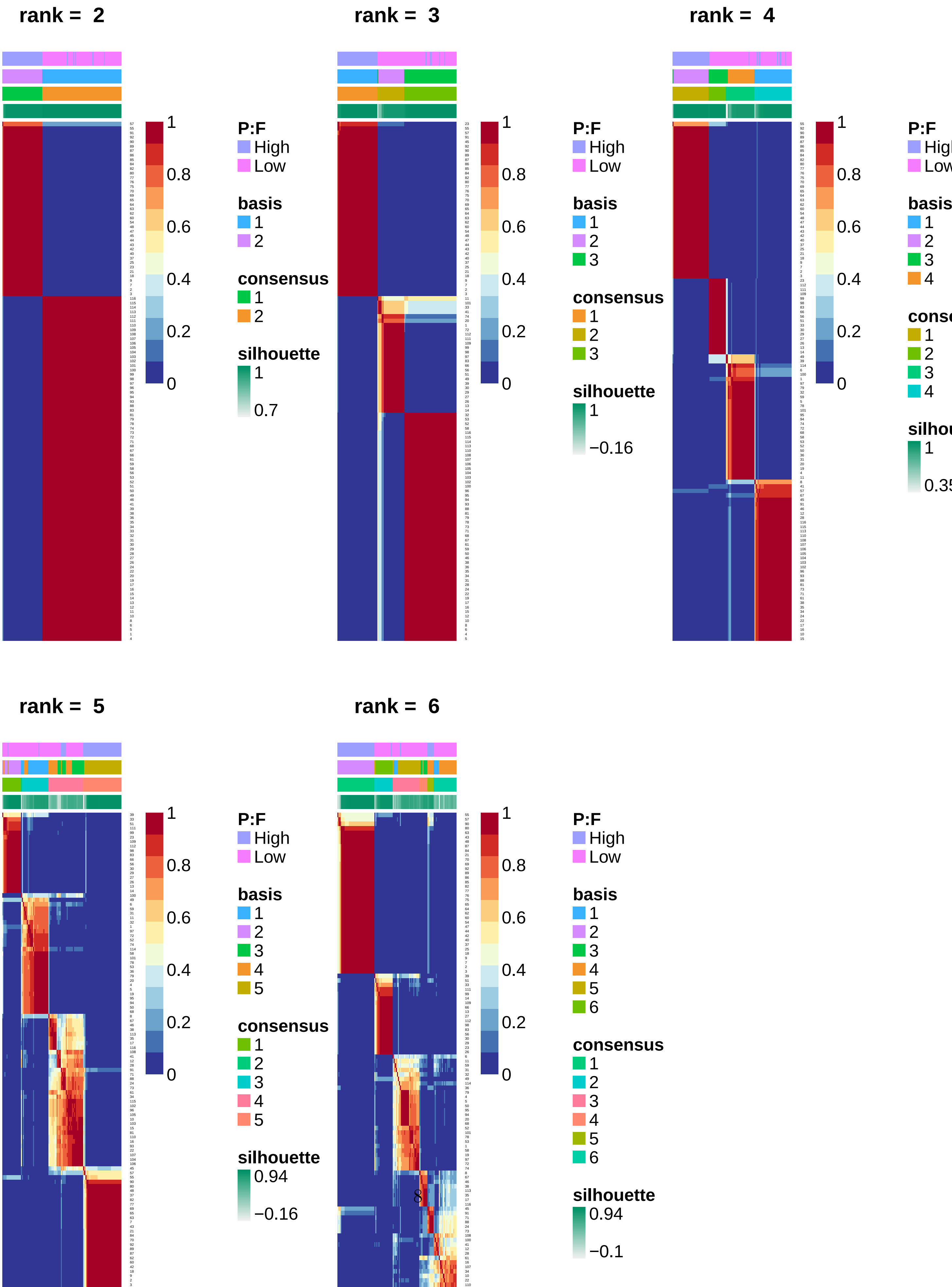
rank = 2
1
0.8
0.6
0.4
0.2
0
P:F
High
Low
basis
1
2
consensus
1
2
silhouette
1
0.7
rank = 3
1
0.8
0.6
0.4
0.2
0
P:F
High
Low
basis
1
2
3
consensus
1
2
3
silhouette
1
−0.16
rank = 4
1
0.8
0.6
0.4
0.2
0
P:F
High
Low
basis
1
2
3
4
1
2
3
4
1
0.35
rank = 5
1
0.8
0.6
0.4
0.2
0
P:F
High
Low
basis
1
2
3
4
5
consensus
1
2
3
4
5
silhouette
0.94
−0.16
rank = 6
1
0.8
0.6
0.4
0.2
0
P:F
High
Low
basis
1
2
3
4
5
6
consensus
1
2
3
4
5
6
silhouette
0.94
−0.1

Table 2: Cluster Concordance

	1	2
1	5	39
2	72	0

Cohen's Kappa for 2 Raters (Weights: unweighted)
Subjects = 116 Raters = 2 Kappa = 0.906
z = 9.81 p-value = 0
