# Supplementary figures and images for: A comprehensive analysis of breast cancer microbiota and host gene expression
Source: PLoS One. 2017 Nov 30;12(11):e0188873. doi: 10.1371/journal.pone.0188873 (PMC5708741; doi:10.1371/journal.pone.0188873)

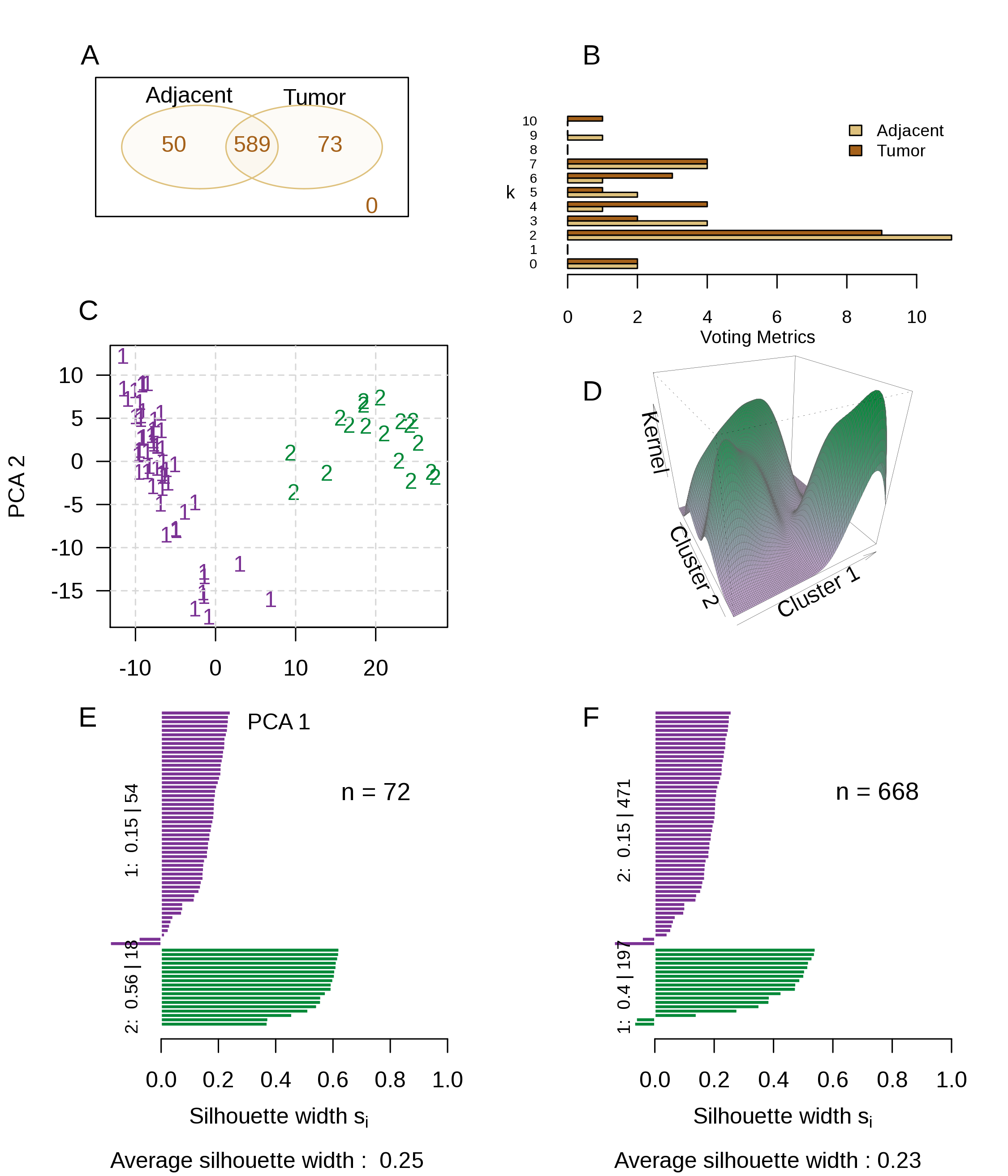

Supplement: S1 Fig — (A) Venn diagram of the bacterial OTU’s observed among the tumors and NCA, demonstrating similar microbial presence. (B) Optimal k-cluster selection analysis demonstrating a majority decision among the 26 metrics for 2 clusters was concordant for both tissue cohorts. (C) A PCA plot of NCA tissues demonstrating separation and cohesion among the smaller second cluster of samples. (D) A perspective plot of the tumor samples demonstrating the sharpness and isolation of tumor samples designated as tumor cluster 1. (E) Silhouette plot for the NCA samples demonstrate a significant proportion, (normal cluster 2, 18 of 72, 0.56) present a distinct batch processing factor which needs to be accounted for. (F) Similarly, the silhouette plot among the tumor samples also demonstrates that a significant proportion (tumor cluster 1,197 of 668, 0.4) also need to be accounted for. (TIF) [file pone.0188873.s004.tif]

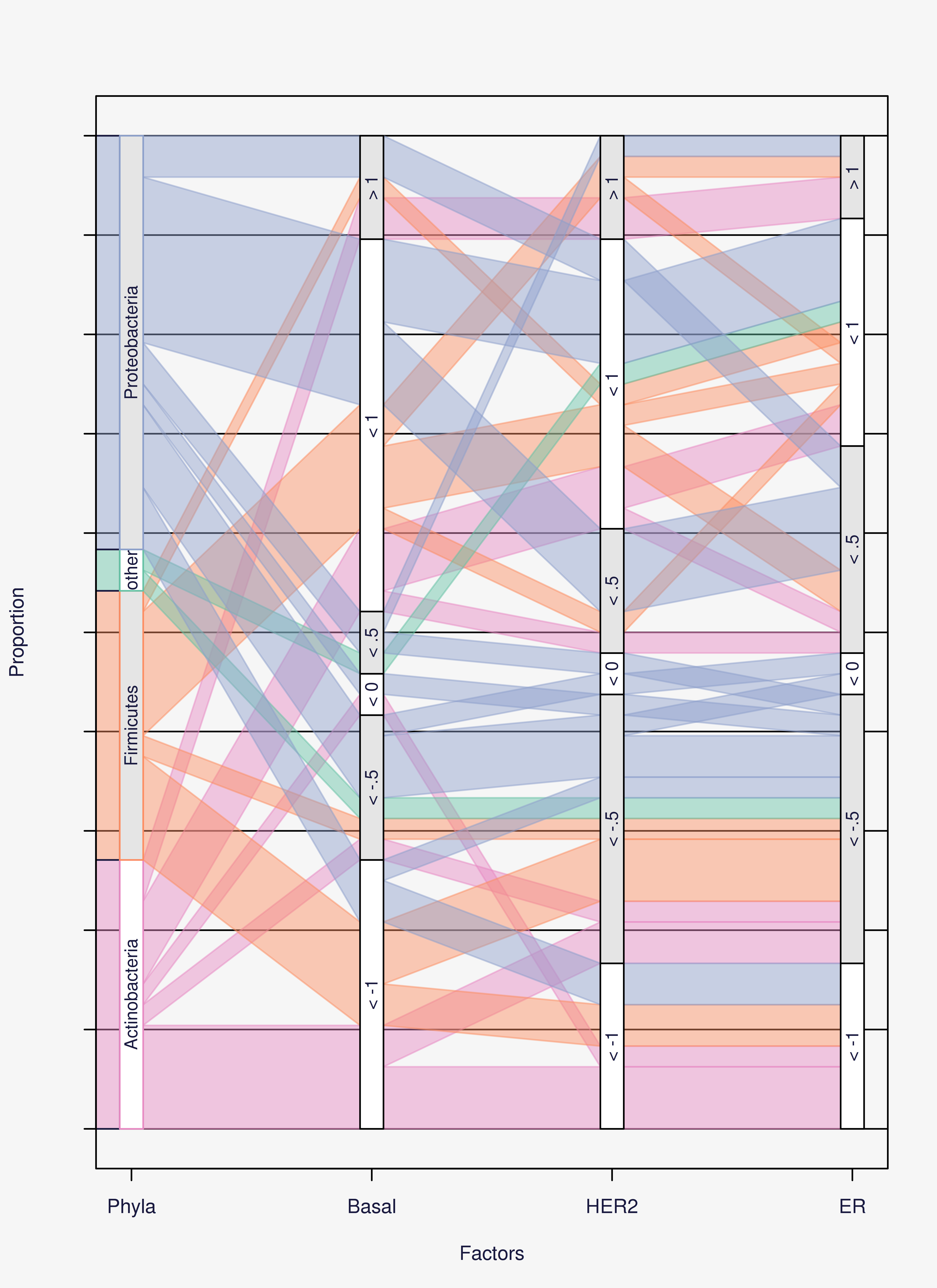

Supplement: S2 Fig — The observed log fold changes were binned for each of the subtype comparison again NCA tissues. (TIF) [file pone.0188873.s005.tif]
